# Prevalence of Caries among School Children in Saudi Arabia: A Meta-Analysis

**DOI:** 10.1155/2022/7132681

**Published:** 2022-09-05

**Authors:** Tasneem R. Adam, Abdullah I. Al-Sharif, Aretas Tonouhewa, Abdulaziz A. AlKheraif

**Affiliations:** ^1^Alfaisal University, College of Medicine, Riyadh, Saudi Arabia; ^2^Research Unit on Communicable Diseases, Abomey-calavi University, 01 BP 2009, Cotonou, Benin; ^3^Dental Biomaterials Research Chair, Dental Health Department, College of Applied Medical Sciences, King Saud University, Riyadh, Saudi Arabia

## Abstract

**Introduction:**

Children's dental health has become the primary concern, because of the increase in the prevalence of caries amongst school children in Saudi Arabia. Therefore, a meta-analysis was conducted to assess the prevalence and severity of dental caries among school children in Saudi Arabia.

**Method:**

A systematic search of Scopus, ISI Web of Science, EMBASE, Saudi digital library, Google Scholar, and MEDLINE via Ovid for cross-sectional studies with healthy participants between 5and –15 years. Two authors independently extracted the prevalence of caries. With 95% confidence intervals (CIs) using a random-effects model, we calculated caries prevalence.

**Results:**

Dental caries prevalence data were extracted from 18 cross-sectional studies (*n* = 56,327 children). The pooled estimate for the caries prevalence among 5–7 years' children was 84% (95% CI: 0.81–0.87%; I^2^ = 91%) while among 12–15 years' children was 72% (95% CI: 0.63–0.79; *I*^2^ = 96.2%). *Discussion*. In this systematic review, the summary estimate of the prevalence of dental caries among children of 5–7 years and 12–15 years were 84% and 72%, respectively. Further research is required to identify approaches for preventing and treating dental caries in schoolchildren.

## 1. Introduction

One of the oldest and most common human infections is dental caries [1]. As shown by the American Dental Association, dental caries can be characterized as a “biofilm-mediated, sugar-driven, multifactorial, and dynamic disease that results in the phasic demineralization and remineralization of dental hard tissues.” [2]. Over the most recent couple of decades, various measures have been executed to prevent dental caries and other oral health problems in many populations over the world. Despite the tremendous endeavours made, it is still a worldwide public health problem and influences an enormous piece of the total population [3]. Although dental caries is preventable, the prevalence remains high in school-age children [4]. In 2010, this infection influenced 2.43 billion people (35.3) % of the population worldwide [5].

A decrease in caries prevalence is seen mainly in nations that have established public health programs using school health programs and school sealant programs to prevent dental caries. The United Kingdom (UK) used to witness high rates of dental caries. However, by applying the oral health framework, the rates started to significantly change the prevalence of oral health in general and dental caries in particular [6]. A British study of 69,318 children between the age of 5–15 years uncovered a 31–51% decrease in caries over the last 40 years (1973–2013) [7]. It appears that these figures have improved with time. In 2019, a study conducted in the UK reported that the prevalence of dental caries ranged from 25–26% among five-year-old children [8].

Among Saudi Arabia (SA) regions, the dental caries prevalence was much higher than in European countries. Since the implementation of the well-being framework. The prevalence of dental caries is high in Saudi populations, especially among young people [9]. Nationally, the prevalence of caries among children between the ages of 6 and 7 was 74% to 90% in the primary teeth. While in permanent teeth, it was 59 80% [10]. The state of caries can be found in research that examined the national prevalence of dental caries in school children in SA. The primary national investigation was done by Al-Shammery (1999) evaluated 1873 children at 12 to 13-year-olds in ten regulatory locales in SA. The article found that the prevalence of caries was 74% in urban areas and 67% in rural areas. The examination discovered a factually massive contrast between the metropolitan regions and the rural zones [6].

It is worth mentioning that in 1993 SA started to apply different prevention measures in primary care centers. In 2005, the first national program that covers all primary schools was launched and ever since the MOH has been providing oral health promotion activities in schools. However, there is no research that provides a true estimate of the prevalence of dental caries in all SA regions to estimate the changes that have occurred. Therefore, this study aims to assess the prevalence and severity of dental caries among schoolchildren in Saudi Arabia.

## 2. Methodology

We report and record this manuscript in accordance Preferred Reporting Items for Systematic Reviews and Meta-analysis (PRISMA statement) guideline [11]. The study protocol was registered after the initial screening stage (ID CRD42020154732).

### 2.1. Eligibility Criteria

The inclusion criteria were as follows: (a) participants between the age of 5 and 15 years (as WHO recommended) [12] who are generally healthy without systemic disease; (b) studies reporting the prevalence of dental caries using the WHO criteria for caries diagnosis; (c) studies in English or Arabic and published between January 1996 and October 2019; and (d) population-based studies conducted in Saudi Arabia. We exclude all the articles that did not completely comply with the inclusion criteria.

### 2.2. Search Strategy

The following databases were thoroughly searched for studies that met the inclusion criteria: Scopus, Google Scholar, ISI Web of Science, and MEDLINE via Ovid. For unpublished studies and theses, TA searched the local databases and journals through the Saudi Digital Library. Refer to Appendices A for detailed information about each database's search strategy. TA and SN reviewed the references of included studies to find relevant papers. The citations that have been identified through the search are imported into Endnote X8.

### 2.3. Data Extraction and Assessment of the Risk of Bias

A data extraction form has been used to extract data from all included studies systematically. Two authors independently extracted the data from the selected studies (TA and SN). Disagreements were settled by the third author (AA). Information extracted in the present review included: author's name; year of the examination; the age of participants study configuration; considered site; sample size; analytic measures; dental caries prevalence; and severity of dental caries (dmft/DMFT list). As per the Cochrane Reviewers' Handbook [13], the risk of bias inside investigations was evaluated freely by two authors (TA and SN). The Newcastle–Ottawa Scale for quality assessments for cross-sectional investigations assessed the methodology for the included examinations [14, 15]. The risk of bias tool comprises five items. Each investigation was ordered as having either a high (score <3) or low (score 3–5) risk of bias [16].

### 2.4. Statistical Methodology

Meta-analysis was performed using R Software v 4.0.5 with package meta 4.15–1 and metafor 2.4–0 according to Schwarzer [17] and Vechtbauer [18]. We have used a random intercept logistic regression model and generalized linear models to pool prevalence estimate as well as DMFT/dmft estimate. Heterogeneity between studies was evaluated by *I*^2^ and Cochrane's Q statistic [19, 20]. A level of 75% and more indicates a high degree of heterogeneity. Publication bias was assessed by inspection of funnel plots and formal Egger's asymmetric test [21]. A *p* value <0.5 on Egger's test will be considered indicative of a statistically significant publication bias.

## 3. Results

### 3.1. Study Selection and Characteristics of the Included Studies

**Figure 1:**
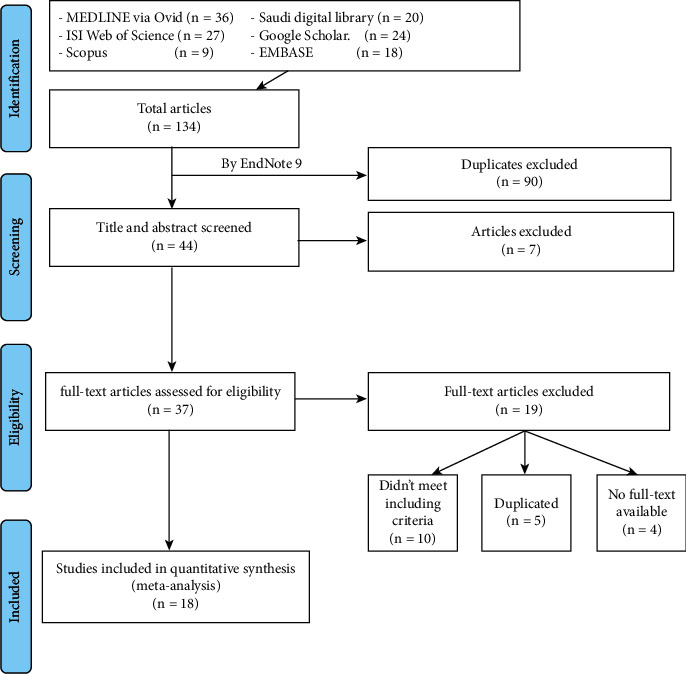
Flowchart illustrates a summary of the literature review and research selection process.

The initial search yielded 134 publications through six different databases. After removing duplicates by EndNote and screening the titles or abstracts, 37 full-text articles were reviewed. Of these, 19 studies were excluded. The remaining 18 studies [5–8, 16, 22–34] (56,327children) were included in this meta-analysis (Flowchart-Figure 1). Included articles were published between 1996 and 2019 with sample sizes ranging from 103 to 39,296. Among the included studies, six were performed in the Riyadh region, four were conducted in the Makkah region, three were conducted in the eastern region, three were performed in the Medina region, and only one study was conducted in the Asser. Caries assessment was conducted through clinical examinations, following WHO criteria (Table 1).

Based on the modified Newcastle–Ottawa Scale (NOS) scale for observational studies, we assessed the methodological quality of included studies, in which studies could obtain up to 5 potential points. Six studies received 5 points, three studies received 4 points, eight studies received 3 points, and one study received 2 points (Table 2).

### 3.2. Prevalence of Dental Caries in the Primary Dentition

**Figure 2:**
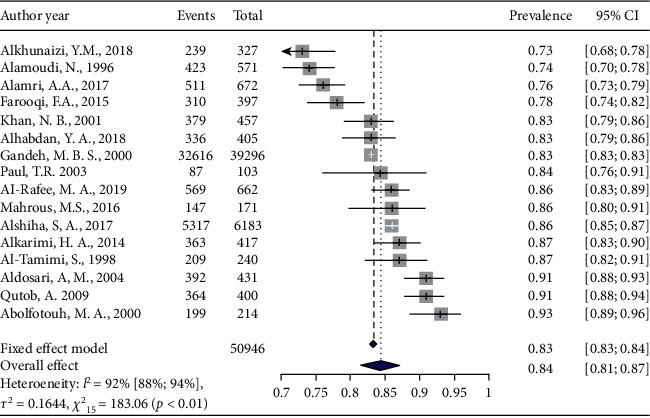
Pooled prevalence of dental caries among 5–7 -year children.

The general prevalence of dental caries among aged 5–7 children was 84% (95% CI: 0.81–0.87% *I*^2^ = 91%) based on the random-effects model. The highest and lowest prevalence of dental caries was observed in different regions of SA was seen in Abha (93%) and Eastern Province (73%), respectively (Figure 2).

### 3.3. Prevalence of Dental Caries in the Permanent Dentition

**Figure 3:**
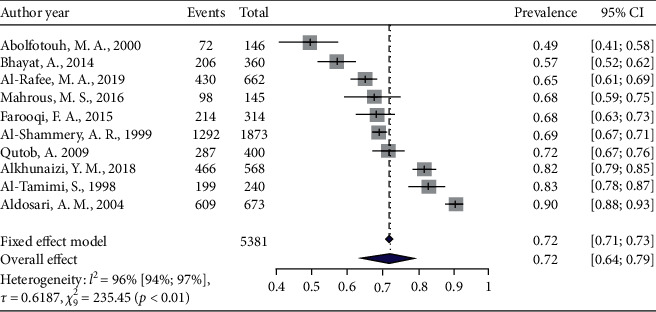
Pooled prevalence of dental caries among 12–15-year children.

The general prevalence of dental caries among 12–15 children was 72% (95% CI: 0.63–0.79%; *I*^2^ = 96%) based on the random-effects model. The maximum and minimum prevalence of dental caries was observed in Riyadh (92.3%) and Abha (52%), respectively. Out of the nine studies, this analysis showed a significant heterogeneity among the studies (Figure 3).

### 3.4. Assessment of Publication Bias

**Figure 4:**
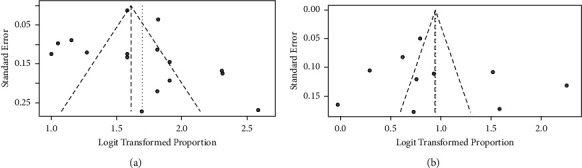
(a): Funnel plot for assessing publication bias for studies that caries prevalence in primary teeth reported. (b): funnel plot for assessing publication bias for studies that caries prevalence in permanent teeth reported.

The symmetry of the Funnel plots and Egger's regression intercept test revealed no indication of publication bias (Figures 4(a) and 4(b)).

## 4. Discussion

This systematic review comprehensively describes the prevalence of dental caries in children living in SA regions based on data published between 1996 and 2019. In this process, data from 18 studies (out of 134 published papers) were considered for quantitative analysis. This review found high prevalence estimates of dental caries and an increase in caries prevalence in the last decade. Our results showed that the prevalence of dental caries was 84 per cent and 72 per cent, respectively, in primary and permanent dentition. Consistent with AlDosari et al., the findings indicate that the prevalence of caries was between 74% and 90% in primary teeth. [10]. These findings are worrisome in the efforts made by the Ministry of Health (MOH) to reduce these figures.

The results have shown that dental caries is still a significant issue in SA despite the government's and dental healthcare workers' attempts to improve oral health in children and increase people's awareness of oral health over the years. In Riyadh, the capital of SA, the present review showed an improvement in the state of caries in schoolchildren compared to the results of the cross-sectional study conducted in the Central Province in 2004 [16]. The situation of caries among children aged 5–7 years in Jeddah has deteriorated in recent years compared to the previous survey in 1996 [27].

In a previous review, Al-Ansari concluded that most studies found that caries has a high prevalence among SA regions [35]. However, some of these papers did not evaluate caries in general populations. Instead, they attempted to estimate the prevalence of caries in participants with systemic disease or special needs. These diseases may have a significant influence on oral health. Nevertheless, we excluded the articles that evaluated caries in a specific population because participants with the systemic disease have a much higher chance of having caries, which does not reflect the true prevalence of the disease.

A systematic review by Alayyan 2017 reported that caries prevalence was 64% in permanent teeth in gulf cooperation council states [36]. However, this review had obvious weaknesses, which are as follows. First, the study missed three important studies: Aboalfotoh 2000, Altamimi 1998 and Qutob 2009 [22, 25, 28]. Each of these articles showed a high level of caries rates, except for Abolfotouh 2000; therefore, incomplete data rendered the results of Alayyan 2017 inaccurate. Besides, Alayyan 2017 inappropriately compared studies conducted among 6–7 old children aimed to measure caries in permanent teeth with other studies done among children aged 11–16 years old [37]. As WHO stated, caries can be measured in primary teeth in children aged 5–7 years. This age is significant in terms of the prevalence of caries in primary dentition, which may change more quickly than permanent teeth [12]. Therefore, it is not logical to measure caries rates in permanent teeth, as they just recently erupted and have not been exposed enough to the oral environment. Nevertheless, the validity of this paper was severely damaged by not mentioning the eligibility criteria for including studies [36].

Another meta-analysis by Alayyan et al. 2018 aimed to evaluate the prevalence and severity of dental caries in preschool children [12]. The review included a study on Bedouin children who live in the desert near the Al-Qasseem region, where it is very difficult to consume sugar in general. Furthermore, the Al-Qasseem region is well known for high levels of fluoride in its water [16, 38]. However, Alayyan 2018 included a study conducted in a hospital setting among 5-12-year-old children [39]. As a 2010 study stated, only 8.6% of the student visited dentists for regular check-ups. While almost half of the students visited the dentist when they have pain or dental problems [40], thus it is obvious that caries prevalence will be very high. As expected, Al-banyan 2001 reported that 99% of children had caries [39].

The World Dental Federation, the WHO, and the International Dental Research Association have worked to plan the Global Oral Health Goals for 2020. One objective was to reduce the impact of dental caries on individuals and society and develop an early diagnosis, prevention, and effective management strategies for dental caries [41]. Unfortunately, most epidemiological studies have shown that dental caries remain prevalent among school children in SA. Also, untreated caries in young children is still a significant health burden in SA, suggesting that more extraordinary efforts and different preventive measures are needed if this goal is to be achieved. In 2007, the Saudi Arabian Standards Organization (SASO) adopted the MOH recommendations regarding fluoride ions to primary drinking water networks in major cities in order to reduce caries prevalence from 90% to 50–60% [42]. However, the findings of this meta-analysis showed that the MOH's goal was not yet met.

This review found that the prevalence of caries in 6-year-old children was higher than in 12-year-old children. In agreement with our results, a 2019 study conducted in Riyadh showed that caries prevalence in primary and permanent teeth was 86% and 65%, respectively [31]. Another study conducted in the same age group but in Jeddah found a similar result, 83.5% for primary teeth and 72% for permanent teeth [28]. These findings are similar to a 2018 study, which showed that caries prevalence remains high among six-year-old children [5]. The increase in the prevalence of caries is consistent with previous findings. A review showed that the prevalence of caries increased between 1985 and 2010 [35].

Caries rates have altered dramatically globally during the last three decades. The prevalence of dental caries in children has decreased in several developed countries using preventive oral care programs. The UK and Sweden had the highest reduction rates in the prevalence of caries (46% and 45%, respectively) over 40 years [3]. The proportions of caries reported in the review indicate that while there appears to be some decrease in the overall prevalence relative to previous national surveys, the changes are not marked as shown in other developed nations. It is likely due to the nature of dental caries; there are multiple factors that play a role in the increase of caries, including lack of awareness, poor oral hygiene, insufficient preventive dental services, and underutilization of dental services [31, 43]. Most studies did not find a relationship between dental caries and socioeconomic state; however, they found a potential correlation between sugar consumption as well as parents' awareness [6, 22, 44]. As for fluoride levels, a cross-sectional study found the levels of fluoride in Riyadh and Qaseem were very high yet did not find a correlation between the water fluoride level and caries experience among school children [16].

Most of the previous studies mentioned the importance of school programs and improving the quality of care of professionals. The MOH indeed has done many campaigns and programs to reduce the prevalence and severity of caries. One of these programs is the National Initiative to Prevent Dental Caries, launched in 2018, which is the largest and most comprehensive campaign. According to MOH, the number of beneficiaries of the initiative reached nearly one million children in 2018 [45]. Nevertheless, there is no study to evaluate the effectiveness of these programs.

The NOS for cross-sectional study quality assessment was used to assess the quality of the included papers in this meta-analysis. Overall, a good number of quality studies about caries prevalence among school children were found mostly from Riyadh, Jeddah, and Medina. The high-quality appraisal scores are due to efficiencies in the methods and reporting of the studies included in this review. Most studies with satisfactory results included large sample sizes and detailed descriptions of the diagnostic criteria utilized. Furthermore, all included investigations used validated methods to measure dental caries.

Our meta-analysis results should be interpreted considering certain limitations. First, there was considerable heterogeneity between studies, although we have established strict inclusion and exclusion criteria. High heterogeneity between studies requires cautious interpretation of the results. The significant heterogeneity of the results could be due to geographical variation and ethnic backgrounds. Second, most studies did not adequately control confounding factors such as fluoride exposure, socioeconomic level, and parental educational level. Finally, since this meta-analysis included only cross-sectional studies, we cannot establish causality.

The present meta-analysis quantified the prevalence of caries among schoolchildren in SA. The statistical analysis showed that there was no publication bias among the included articles. However, the results of this study should interpret with caution due to the high heterogeneity between the included studies.

The current review discoveries propose that dental caries influence a significant part of young students in SA. Given the multifactorial idea of dental caries, various methodologies should be taken next to each other to roll out an exceptional improvement. Creating a national oral health policy that emphasizes the promotion and prevention of oral health would be most beneficial for addressing oral health problems in school children in Saudi Arabia. In schools, oral health education should be combined with the school curriculum. Previous studies recommended that early dental health education should focus on teaching pregnant and nursing mothers as well as caretakers. Furthermore, educational messaging should take into account the parents' socioeconomic situation and be attentive to cultural differences. This review may help public health managers, policymakers, and stakeholders evaluate such projects and may serve as a baseline for future national studies.

## 5. Conclusion

In this systematic review, the summary estimate of dental caries prevalence among 5-7 years-old and 12–15 years-old children were 84% and 74%, respectively. We conclude that dental caries status among school children is high in SA. Further research is required to identify approaches for preventing and treating dental caries in schoolchildren. Further studies on the ongoing programs to evaluate the effectiveness of oral health promotion programs are essential.

## Figures and Tables

**Table 1:** Characteristics of included studies.

Author and year	Study location	Age group	Gender	Sample size	Dental caries measurement	Prevalence of dental caries
Al‐Tamimi, S., 1998	Medina	6, 12	Both	480	DMFT + dmft	Primary teeth 87% Permanent teeth 83%
Bhayat, A., 2014	Medina	12	Male	360	DMFT	Primary teeth 57.2%
Mahrous, M. S., 2016	Medina	6, 12	Both	316	DMFT + dmft	Primary teeth 86% Permanent teeth 67.6%
Abolfotouh, M. A., 2000	Abha	6, 12	Male	959	DMFT + dmft	Primary teeth 93.3% Permanent teeth 49.5%
Alkhunaizi, Y. M., 2018	Dhahran, Al-Khobar	6, 12, 15	Female	895	DMFT + dmft	Primary teeth 73% Permanent teeth 82%
Farooqi, F. A., 2015	Dammam	6, 7, 12	Both	711	DMFT + dmft	Primary teeth 77.8% Permanent teeth 68%
Khan, N. B., 2001	Al-ahsa	6–7	Both	457	dmft	Primary teeth 82.9%
Alamoudi, N., 1996	Jeddah	6, 7	Not reported	571	dmft	Primary teeth 73.5%
Qutob, A. (2009)	Jeddah	6, 12	Both	800	DMFT + dmft	Primary teeth 91% Permanent teeth 71.7%
Gandeh, M. B. S., (2000)	Jeddah	6–7	Both	39,296	dmft	Primary teeth 83.4%
Alkarimi, H. A., 2014	Jeddah	Mean age 6.8	Both	417	dmft	Primary teeth 87.1%
Al-rafee, M. A., 2019	Riyadh region	6, 12, 15	Both	1986	DMFT + dmft	Primary teeth 85.77% Permanent teeth 64.98%
Aldosari, A. M., 2004	Riyadh and qaseem	6–7 and 12–13	Both	1104	DMFT + dmft	Primary teeth 91.2% Permanent teeth 90.5%
Alamri, A. A., 2017	Riyadh	6	Male	672	dmft	Primary teeth 76.4%
Alshiha, S. A., 2017	Riyadh	6–7	Female	6183	dmft	Primary teeth 85.5%
Alhabdan, Y. A., 2018	Riyadh	Mean age 6.92	Male	578	dmft	Primary teeth 83%
Paul, T. R. 2003	Al-kharj	5	Both	103	dmft	Primary teeth 83.5%
Al‐Shammery, A. R., 1999	Saudi Arabia	12–13	Both	1873	DMFT	Rural areas were 67% Urban areas were 74%

**Table 2:** Modified newcastle-ottawa risk of bias score for the 20 studies included in this systematic review and meta-analysis.

Study	Sample representativeness	Sample size	Response rate	Ascertainment of dental caries^*∗*^	Descriptive statistics	Total
Al-tamimi, S., 1998			^*∗*^	^*∗*^	Didn't mention confidence intervals or p-value	**4**
Bhayat, A., 2014	Only boys	^*∗*^		^*∗*^		**3**
Mahrous, M. S., 2016	^*∗*^		^*∗*^	^*∗*^	^*∗*^	**5**
Abolfotouh, M. A., 2000	Only boys	^*∗*^	^*∗*^	^*∗*^	^*∗*^	**5**
Alkhunaizi, Y. M., 2018	Only female	^*∗*^		^*∗*^	Didn't mention confidence intervals or p-value	**3**
Farooqi, F. A., 2015	^*∗*^	Didn't justify the sample size		^*∗*^	^*∗*^	**3**
Khan, N. B., 2001	^*∗*^	^*∗*^		^*∗*^		**3**
Alamoudi, N., 1996	^*∗*^	^*∗*^	Didn't mention the number of consent form	^*∗*^		**3**
Qutob, A. 2009	^*∗*^	^*∗*^	^*∗*^	^*∗*^	^*∗*^	**5**
Gandeh, M. B. S., 2000	^*∗*^	^*∗*^		^*∗*^		**3**
Alkarimi, H. A., 2014		Didn't justify the sample size	^*∗*^	^*∗*^	^*∗*^	**3**
Al-rafee, M. A., 2019	^*∗*^	^*∗*^	^*∗*^	^*∗*^	^*∗*^	**5**
Aldosari, A. M., 2004	^*∗*^	^*∗*^		^*∗*^		**3**
Alamri, A. A., 2017	Only boys	^*∗*^		^*∗*^	^*∗*^	**4**
Alshiha, S. A., 2017	Only female	^*∗*^	^*∗*^	^*∗*^	^*∗*^	**5**
Alhabdan, Y. A., 2018	Only boys	^*∗*^	^*∗*^	^*∗*^	^*∗*^	**4**
Paul, T. R. 2003				^*∗*^	^*∗*^	**2**
Al-Shammery, A. R., 1999	^*∗*^	^*∗*^	^*∗*^	^*∗*^	^*∗*^	**5**

^*∗*^All the included studies used the WHO diagnostic criteria.

## Data Availability

All data generated or analysed during this study are included in this manuscript (and its supplementary information files).

## Supplementary Materials

Supplementary Materials Detailed information about each database's search strategy.
