# Supplementary material for: Prevalence of Caries among School Children in Saudi Arabia: A Meta-Analysis
Source: Adv Prev Med. 2022 Sep 5;2022:7132681. doi: 10.1155/2022/7132681 (PMC9467753; doi:10.1155/2022/7132681)
Supplement: Supplementary Materials — Detailed information about each database's search strategy. [file 7132681.f1.docx]

**Appendice**

**Search strategy for electronic databases**

*Search strategy for identification of studies*

Studies were identified by electronic searches of Scopus, ISI Web of Science, EMBASE, Saudi digital library, Google Scholar, and MEDLINE via Ovid.

**The following strategy was used to search**

**Scopus** [search conducted November 22, 2019]:

( children OR child OR student OR school OR schoolchildren ) AND ( caries OR intracavity OR carious OR decay ) AND ( saudi OR arabia OR riyadh OR Jeddah OR dammam ) AND ( prevalence OR epidemiology OR epidemiologic ) ( LIMIT-TO ( LANGUAGE , "English" ) )

**ISI Web of Science** [search conducted November 23, 2019]:

#1 (ALL=(Prevalence OR oral health survey OR surveys OR Cross-sectional studies OR Epidemiology OR Epidemiologic OR Epidemiologic OR Epidemiologic studies OR Epidemiologic measurements)) AND LANGUAGE: (English)

#2 (ALL=(Dental caries OR Caries OR Dentalcavity OR dental cavity OR Carious OR Decay OR tooth decay)) AND LANGUAGE: (English)

#3 (ALL=(Saudi Arabia OR Saudi OR Arabia OR Riyadh OR Jeddah OR Dammam)) AND LANGUAGE: (English)

#4 (#1 AND #2 AND #3)

#5 (ALL=(Children OR Child OR student OR Primary teeth OR permanent teeth OR school OR schoolchildren)) AND LANGUAGE: (English)

5# #4 AND #5

**MEDLINE** via ovid search strategy [search conducted November 24, 2019]

#1 exp Child/
#2 student.mp.
#3 (school or children or school-children).mp.
#4 1 or 2 or 3
#5  exp Dental Caries
#6  (caries or cavity or Carious or decay).mp.
#7  caries$.mp.
#8  4 and 6 and 7
#9  exp Prevalence/
#10 oral health survey.mp.
#11 Epidemiology$.mp.
#12  9 or 10 or 11
#13  8 and 12
#14  exp Saudi Arabia/
#15  Saudi$.mp.
#16  riyadh.mp.
#17  Jeddah.mp.
#18 (14 or 15 or 16 or 17)
#19  8 and 13 and 18

**EMBASE** via Ovid search strategy [search conducted November 26, 2019]:

#1. child$.ti,ab.

#2. student$.ti,ab.

#3. (school$ or children$ or school-children$).ti,ab.

#4. dental caries$.ti,ab.

#7. caries or cavity or Carious or decay$.ti,ab.

#8. caries$.ti,ab.

#9. 4 and 6 and 7

#10. exp Prevalence/

#11. oral health survey.mp.

#12 Epidemiology$.mp.

#13 9 or 10 or 11

#14 8 and 12

#15 exp Saudi Arabia/

#16 Saudi$.mp.

#17 Arabia$.mp.

#18 riyadh$.mp.

#19 Makkah$.mp.

#20 Eastern$.mp.

#21 (16 or 17 or 18 or 19 or 20)

#22 9 and 14 and 22

**Saudi digital library** search strategy [search conducted November 27, 2019]

1 ALL=(Prevalence OR oral health survey OR surveys OR Cross-sectional studies OR Epidemiology OR Epidemiologic OR Epidemiologic studies OR Epidemiologic measurements)

2 ALL=(Dental caries OR Caries OR Dentalcavity OR dental cavity OR Carious OR Decay OR tooth decay)

3 #2 AND #1

4 ALL=(Saudi Arabia OR Saudi OR Arabia OR riyadh OR Jeddah OR Dammam

5 ALL=(Children OR Child OR student OR Primary teeth OR permanent teeth OR school OR schoolchildren

6 #5 AND #4

7 #6 AND #3

8 ALL=(preschool OR preschool children)

9 #7 NOT #8

10 (#9) AND LANGUAGE: (English)

Refined by: ORGANIZATIONS-ENHANCED: ( KING SAUD UNIVERSITY OR KING ABDULAZIZ UNIVERSITY OR IMAM ABDULRAHMAN BIN FAISAL UNIVERSITY OR KING SAUD BIN ABDULAZIZ UNIVERSITY FOR HEALTH SCIENCES OR TAIBAH UNIVERSITY )

**Google Scholar** search strategy [search conducted November 28, 2019]

“Children” or “schoolchildren” and “Carious” or “Caries” and “Prevalence” or “Epidemiology” and “Saudi” -malocclusion -India -fluorosis -gingivitis
